# Chronic Kidney Disease-Epidemiology Collaboration (CKD - EPI) classification of kidney function and predictors of kidney dysfunction among type 2 diabetes mellitus patients in a tertiary hospital in Ghana

**DOI:** 10.11604/pamj.2024.49.132.43686

**Published:** 2024-12-20

**Authors:** Godsway Edem Kpene, Enoch Kwame Obuobi, Gifty Dzifa Aku Senoo, Priscilla Appiah Baffoe, Georgina Korankye

**Affiliations:** 1 Department of Medical Laboratory Sciences, School of Allied Health Sciences, University of Health and Allied Sciences, Ho, Ghana

**Keywords:** Type 2 diabetes mellitus, kidney, kidney function, Ghana, predictors

## Abstract

**Introduction:**

Type 2 Diabetes Mellitus (T2DM) is a major global health concern frequently associated with Kidney Dysfunction (KD). Globally, approximately one in eleven adults have diabetes mellitus, with 90% of these cases being type 2 diabetes mellitus. About two-thirds of T2DM patients experience KD, which may progress to chronic kidney disease and end-stage renal disease. In Ghana, the burden of T2DM is substantial and continues to rise, with kidney failure accounting for approximately 10% of all deaths among individuals with T2DM. This study sought to investigate the predictors of KD among T2DM patients in Ghana and approximately 10% of deaths in people with T2DM are attributable to kidney failure.

**Methods:**

a hospital-based retrospective study design was employed. It involved the medical records of 141 T2DM patients. The data extracted was entered into Microsoft Excel version 16.0 and analyzed using STATA version 16.0. Chi-square test was used to establish associations between categorical variables and KD. Independent t-test was employed to analyze associations between parametric (normally distributed) variables and KD, while the Mann-Whitney U test was used for non-parametric (not normally distributed) variables. The strengths of the identified associations were evaluated using binary logistic regression analysis, with the results reported as odds ratios (OR) along with their 95% confidence intervals.

**Results:**

among the 141 patients, 99 (70.2%) had KD. Formal employment was associated with a 95% reduced odds of kidney dysfunction (aOR = 0.05 (95%CI: 0.004-0.645); p-value = 0.021), while every unit increase in creatinine level was linked to a 10% increased odds of KD (aOR = 1.10 (95%CI: 1.06-1.14); p-value = <0.001).

**Conclusion:**

the study revealed a significant proportion of T2DM patients experiencing kidney dysfunction. Crucially, both occupation and creatinine levels were found to be independent predictors of diabetic KD. This highlights an urgent need to educate T2DM patients, particularly those who are unemployed or informally employed, about preventive measures and the importance of regular monitoring of creatinine levels to safeguard kidney health. This emphasis is vital, as managing kidney disease in the region is notably costly, making early intervention and education key strategies in reducing the burden of KD among T2DM patients.

## Introduction

Type 2 diabetes mellitus (T2DM) is the most common form of Diabetes Mellitus (DM) characterized by hyperglycemia, insulin resistance, and relative insulin deficiency [1]. The condition continues to be a global health concern, and as a result of this tendency, it is quickly becoming an epidemic in various nations around the world [2,3]. The incidence of diabetes, which was 10.5% in 2021, was predicted by the International Diabetes Federation to rise to 11.3% by 2030 and 12.2% by 2040 [4]. Based on available statistics, the global diabetes population is estimated to be 540 million, with type 2 diabetes accounting for more than 90% of cases. T2DM is associated with a myriad of complications and despite rates of diabetes-related complications such as cardiovascular disease, retinopathy, and neuropathies decreasing significantly in the past two decades, the same cannot be said of kidney complications [5]. With loss of kidney function (KD) being one of the most common complications of T2DM, about two-thirds of T2DM patients experience KD, which can progress to chronic kidney disease (CKD) and eventually end-stage renal disease (ESRD) [6,7].

Kidney dysfunction is characterized by a sudden decline in renal function, resulting in an inability to secrete waste products and maintain electrolyte and water balance. It is associated with substantial risks of morbidity and mortality [8]. The condition in T2DM not only imposes a substantial healthcare burden but also significantly diminishes the quality of life for affected individuals. Globally, the World Health Organization lists KD as one of the top 10 causes of mortality [9]. It is a significant problem among 20% to 40% of diabetics [10]. Kidney dysfunction in T2DM patients is common in the USA and worldwide [11]. Asian countries have a substantially greater rate of diabetes-related KD than Western nations [12]. Between 2013 and 2016, 36% of diabetic patients in the United States developed diabetic KD [13]. In developing countries, T2DM patients are at a particularly higher risk of developing KD compared to those in developed countries [7] and in the African setting, the prevalence of KD among T2DM patients was found to be 22.0% [14].

In Ghana, the escalating prevalence of T2DM is a significant healthcare challenge and has become a pressing public health concern. The prevalence of CKD among T2DM patients is estimated to be around 27% [15] and approximately 10% of deaths in people with T2DM are attributable to kidney failure [16]. Despite advancements in diabetes management, a significant number of individuals with T2DM continue to develop kidney complications, which can progress to end-stage renal disease. This highlights a critical research gap: the need to identify and understand the predictors of KD among T2DM patients in Ghana. Investigating these predictors is crucial for early detection and prevention, particularly using the CKD-EPI classification equation. This study aims to explore the various factors that may contribute to the development of KD in T2DM patients. By identifying these predictors and clarifying their significance, the study will enhance risk assessment, inform clinical decision-making, and ultimately improve the overall care and quality of life for T2DM patients facing the complex interplay between diabetes and renal function.

## Methods

**Study design:** the study was a hospital-based retrospective study involving the medical records of T2DM patients at the Ho Teaching Hospital (HTH) from January 2017 to November 2022. The retrospective approach was adopted because it is particularly useful for studying rare diseases and outcomes. In the context of T2DM, examining years of medical records makes it possible to identify and analyze less common complications and manifestations like kidney disease that might not be easily captured in shorter, prospective studies [17].

**Study area:** the study was carried out at HTH. The hospital facility is the main referral center in the Volta Region. HTH is the fifth public Teaching Hospital in Ghana and serves the needs of the region and beyond. The Hospital has over 300-bed capacity to cater for the health needs of patients [18].

**Study population:** the study population was all the accessible medical records of T2DM patients 18 years or older who accessed health care at the HTH from January 2017 to November 2022.

### Inclusion and exclusion criteria

**Inclusion criteria:** both available electronic and manual medical records of T2DM patients aged 18 years and above who had complete sociodemographic characteristics data, lifestyle variables (alcohol intake and smoking status), urine protein and glucose results, systolic and diastolic blood pressures as well as creatinine levels within the stipulated period for the study, were included.

**Exclusion criteria:** data on type 1 diabetic patients and patients with T2DM whose data could not be found in the medical records (both electronic and manual) or were incomplete were excluded from the study.

**Sample size:** a complete enumeration was done to collect in-patient type 2 diabetic mellitus data both electronically and manually.

**Data collection and management:** data were extracted from the electronic and manual patient folders using an extraction sheet. The extraction sheet captured data on sociodemographic characteristics, lifestyle (alcohol intake and smoking status), blood pressure (systolic and diastolic), and laboratory estimates (creatinine levels). The resulting data retrieved were coded and cleaned in MS Excel and password protected to ensure data security. All patient entries with missing creatinine levels were deleted. All categorical independent variables were coded numerically and labelled appropriately. Age was categorized into <30 years, 30 - 49 years, 50 - 69 years, and >69 years. The various occupations of the patients were also coded into unemployed, informal, formal, and retired. The dependent variable, kidney function status, was coded as 1 for eGFR of ≤90 ml/min/1.73 m^2^ and 0 for eGFR of >90 ml/min/1.73 m^2^.

**Chronic Kidney Disease Epidemiology Collaboration (CKD-EPI) equation for classifying kidney function:** the CKD-EPI estimate of renal function was calculated as recommended: for women with a plasma creatinine ≤0.7, (plasma creatinine/0.7)^-0.329^X (0.993)^age^(x 166 if black; x 144 if white or other); for women with a plasma creatinine >0.7, (plasma creatinine/0.7)^≤1.209^x (0.993)^age^(x 166 if black; x 144 if white or other); for men with a plasma creatinine ≤0.9; (plasma creatinine/0.9)^-0.411^x (0.993)^age^(x 163 if black; x 141 if white or other); for men with a plasma creatinine >0.9, (plasma creatinine/0.9)^-1.209^x (0.993)^age^(x 166 if black; x 144 if white or other) [19]. An eGFR of >90 ml/min/1.73 m^2^ was considered normal kidney function [20]. The estimated renal functions were expressed in ml/min per 1.73 m^2^.

**Data analysis:** data extracted were exported to STATA version 16.0 for statistical analysis. Quantitative variables were presented as mean ± SD for those that were parametric and median (IQR) for those that were non-parametric. Frequencies and percentages were used to summarize categorical variables. To determine the associations of the study variables with KD, Chi-square test was done for categorical variables while independent t-test (for parametric) and Mann-Whitney U test (for non-parametric) were done for quantitative variables. The strengths of identified associations were further evaluated using binary logistic regression analysis and presented as a crude and adjusted odds ratio. A p-value of 0.05 was considered statistically significant.

**Ethics statement:** the study was approved by the research and ethics committee of Ho Teaching Hospital (ID No: HTH-REC (20) FC_2022). Permission was also sought from the records department of the facility before the start of data collection. To ensure patient confidentiality, all data gathered on the patients during the study were kept confidential to the principal investigator of the study. Unique codes were used for the identification of all study participants rather than their respective names. Under no circumstance was any information retrieved shared with a third party.

## Results

**Figure 1:**
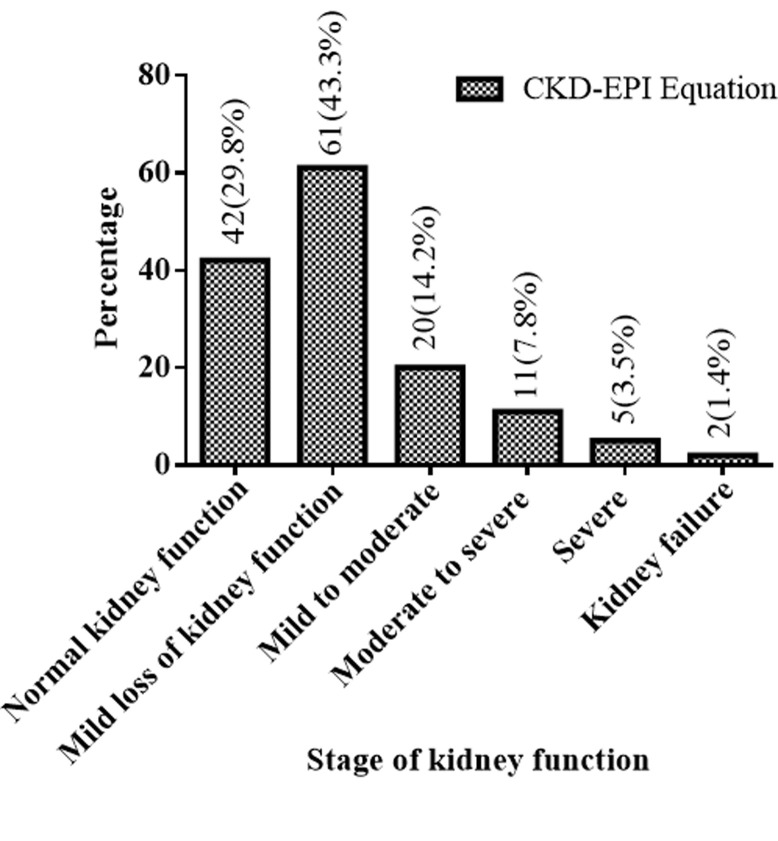
prevalence of kidney function among T2DM patients based on the CKD-EPI equation

Figure 1 shows the prevalence of the various stages of kidney function among the T2DM patients in the study using the CKD-EPI equations. Kidney dysfunction was found among 99 (70.2%) T2DM patients. Specifically, a third of the patients had mild loss of kidney function 61 (43.3%); 5 (3.5%) had severe loss of kidney function while 2 (1.4%) had kidney failure.

Age category and occupation showed significant association with KD. The highest proportion of loss of kidney function was observed among those between 50 - 69 years (79.10%) followed by those older than 69 years (72.22%) (p-value = 0.041). Being retired and unemployed also presented with an increased proportion of loss of kidney function at 90.48% and 78.57% respectively (p-value = 0.035) (Table 1).

**Table 1:** sociodemographic and lifestyle characteristics of participants stratified by kidney function (using CKD-EPI equation)

Variables	Total	CKD - EPI		p-value
		≤90 ml/min/1.73 m^2^	>90 ml/min/1.73 m^2^	
**Age**				**0.041**
<30	8(5.67)	4(50.00)	4(50.00)	
30 - 49	30(21.28)	16(53.33)	14(46.67)	
50 - 69	67(47.52)	53(79.10)	14(20.90)	
>69	36(25.53)	26(72.22)	10(27.78)	
**Sex**				**0.981**
Male	64(45.39)	45(70.31)	19(29.69)	
Female	77(54.61)	54(70.13)	23(29.87)	
**Marital status**				**0.265**
Single	40(28.37)	27(67.50)	13(32.50)	
Married	91(64.54)	63(69.23)	28(30.77)	
Widowed	8(5.67)	8(100.00)	0(0.00)	
Divorced	2(1.42)	1(50.00)	1(50.00)	
**Occupation**				**0.035**
Unemployed	28(19.86)	22(78.57)	6(21.43)	
Informal	70(49.65)	42(60.00)	28(40.00)	
Formal	22(15.60)	16(72.73)	6(27.27)	
Retired	21(14.89)	19(90.48)	2(9.52)	
**Educational status**				**0.836**
None	21(14.89)	13(61.90)	8(38.10)	
Primary	60(42.55)	43(71.67)	17(28.33)	
Secondary	20(14.18)	14(70.00)	6(30.00)	
Tertiary	40(28.37)	29(72.50)	11(27.50)	
**Alcohol intake**				**0.321**
No	110(78.01)	75(68.18)	35(31.82)	
Yes	31(21.99)	24(77.42)	7(22.58)	
**Smoking status**				**0.438**
No	134(95.04)	95(70.90)	39(29.10)	
Yes	7(4.96)	4(57.14)	3(42.86)	
**Location**				**0.594**
Urban	72(51.06)	52(72.22)	20(27.78)	
Rural	69(48.94)	47(68.12)	22(31.88)	

Data is presented in figures and percentages in parentheses. P-value is significant at <0.05

The study observed that those with KD were older (59.44 ± 16.65 years) compared to those with normal kidney function (52.64 ± 16.65 years) (p-value = 0.015). Similarly, the DBP (80.85 ± 18.84 vs 74.05 ± 15.38 mmHg; p-value = 0.041), SBP (134.65 ± 24.10 vs 122.40 ± 27.36 mmHg; p-value = 0.009) and creatinine levels (102.15 (46.49) umol/L vs 67.53 (53.51) umol/L; p-value = <0.001) were higher among patients with loss of kidney function compared to those without (Table 2). After adjusting for all other factors, formal occupation and creatinine levels were independently associated with KD. Being formally employed presented patients with a 95% reduced odds of having KD (aOR = 0.05 (95%CI: 0.004-0.645); p-value = 0.021). Thus, formal employment offers a protective against the development of KD. While each unit increase in creatinine level was associated with a 10% higher odds of developing KD (aOR = 1.10 (95%CI: 1.06-1.14); p-value = <0.001) (Table 3). If a patient's creatinine level rises from 1.0 mg/dL to 2.0 mg/dL, the odds of that patient experiencing KD would increase by 10%.

**Table 2:** biochemical and haemodynamic variables of participants stratified by kidney function (using CKD-EPI equation)

Variables	Total	CKD-EPI equation		
		Normal	≤90 ml/min/1.73 m^2^	p-value
Age (years)	57.42 ± 15.26	52.64 ± 16.65	59.44 ± 16.65	0.015
DBP (mmHg)	80.0 (20.0)	74.05 ± 15.38	80.85 ± 18.84	0.041
SBP (mmHg)	131 ± 25.64	122.40 ± 27.36	134.65 ± 24.10	0.009
*FBG (mmol/L)	9.8 (5.6)	9.9 (7.10)	9.8 (5.55)	0.817
*Cr (umol/L)	94.0 (46.1)	67.53 (53.51)	102.15 (46.49)	<0.001

Data is presented as mean ± SD; *: median (IQR); P-value is significant at <0.05; DBP: dystolic blood pressure; SBP: systolic blood pressure; FBG: fasting blood glucose; Cr: creatinine

**Table 3:** crude and adjusted odd ratio of associated factors to kidney function using the CKD-EPI equation

Variable	cOR	95%CI	p-value	aOR	95%CI	p-value
Age(years)	1.03	1.01-1.06	0.017	1.03	0.92-1.16	0.632
**Age categories (years)**						
<30	1			1		
30 - 49	1.14	0.24-5.44	0.867	1.97	0.04-93.73	0.731
50 - 69	3.79	0.84-17.07	0.083	5.47	0.04-741.66	0.497
>69	2.60	0.54-12.45	0.232	0.93	0.001-637.56	0.983
**Occupation**						
Unemployed	1			1		
Informal	0.41	0.15-1.14	0.086	0.16	0.02-1.14	0.067
Formal	0.73	0.20-2.67	0.632	0.05	0.004-0.645	0.021
Retired	2.59	0.47-14.38	0.276	0.49	0.037-6.359	0.583
SBP (mmHg)	1.02	1.00-1.03	0.011	1.004	0.97-1.04	0.803
DBP (mmHg)	1.02	1.00-1.04	0.042	1.02	0.97-1.08	0.465
Creatinine(umol/L)	1.09	1.05-1.12	<0.001	1.1	1.06-1.14	<0.001

SBP: systolic blood pressure; DBP: diastolic blood pressure; aOR: adjusted odd ratio; cOR: crude odd ratio; P-value is significant at <0.05

## Discussion

The study sought to determine the prevalence of kidney function and predictors of KD among T2DM patients. Like most T2DM-related studies, the current study demonstrated the prevalence of various stages of kidney function spanning from normal kidney function to kidney failure. This study reported a prevalence of 70.2% KD among the T2DM patients. In comparing our findings with that of other studies; we found out that the prevalence of KD was higher in our study compared with the studies by Kim *et al*. [21] and Mhundwa *et al*. [14] who reported 59.5% and 25.0% respectively. Aside from differences in geographical location, sample size, and study design being plausible reasons for these observed variations, the medications used in the management of T2DM could also be accountable. The study by Tsimihodimos *et al*. [22] reported that sodium-glucose cotransporter 2 (SGLT2) inhibitors offer a highly promising treatment option for type 2 diabetes, particularly for patients with established cardiovascular disease. In addition to their glucose-lowering effects, these drugs, along with pioglitazone and incretin mimetics, have shown potential in reducing the incidence and slowing the progression of diabetic nephropathy. Their ability to provide cardiovascular and renal protection makes them valuable additions to the therapeutic arsenal for managing type 2 diabetes and its complications.

The age of patients was a significant predictor of KD in this study. The highest proportion of KD was observed among 50-60-year-olds (79.10%) and those older than 60 years (72.22%) hence attributing that aging influences renal functionality. Although further probing as to why aging among T2DM patients negatively impacts renal health was not established by the current study, the underpinning alterations associated with senescence [23], as well as the overt rise in blood glucose and associated comorbidities (hypertension) [24] reported among diabetics, provides some clarity as to why renal functionality declines with increasing age. The decline in renal function with age is influenced by multiple factors, including structural changes, hemodynamic alterations, oxidative stress, inflammation, mitochondrial dysfunction, hormonal changes, and the accumulation of advanced glycation end products (AGEs) [25,26]. As people age, they naturally lose nephrons and experience glomerulosclerosis and tubular atrophy, which reduce kidney filtering capacity [27]. Decreased renal blood flow and increased arterial stiffness also impair oxygen and nutrient delivery. Chronic low-grade inflammation and oxidative stress can damage renal cells, while mitochondrial dysfunction lowers energy production and raises reactive oxygen species (ROS) levels, leading to cellular damage [28]. Hormonal dysregulation and AGE accumulation further contribute to the stiffening of the glomerular basement membrane and promote fibrosis [29]. These mechanisms collectively result in a natural decline in renal function, which can be worsened by conditions like hypertension, diabetes, and cardiovascular disease.

This study also established that among retirees and the unemployed, there was a significant increase in the number of patients with loss of kidney function. The proportion of 90.48% and 78.57% of patients in the respective categories had KD. Although unaccounted for by the current study, a longitudinal study by Smith *et al*. [30] reporting similar findings discovered that aged T2DM patients in retirement usually have diet and exercise modifications which in turn affect their renal function and glycemic control over time; the latter when poorly done affects tubular and vascular health. One´s occupation also had a significant association with renal outcome. Existing evidence shows that even among people living with clinically established chronic kidney disease, having a job increases their chances of accessing preventive measures that could hinder the further decline in renal function [31]. While being unable to keep a job increases one's risk of renal dysfunction, partly due to lack of a steady income source to purchase medications, low self-esteem as well as other psychological factors.

In addition to the earlier findings presented, this study also investigated blood pressure dynamics which have been implicated by similar studies related to T2DM [32]. The current study, in comparing blood pressure levels among two renal function states; thus, KD and normal kidney function, established elevated blood pressure (diastolic and systolic blood pressures) in T2DM patients with renal impairment compared to those with normal renal activity. Similar reports have been provided by Yu *et al*. [33] regarding increased blood pressure readings and rapid decline of renal function. Our findings can be explained based on the underlying pathophysiological process of hypertension. As a result of fluid and sodium retention in the blood vessels partly due to the inability of the kidneys to rid the body of excess fluids, wastes, or electrolytes. The current finding corroborates with the study by Mulu *et al*. [34] who likewise reported similar blood pressure levels and established statistically significant associations that are fairly similar to that presented in this study (DBP (80.85 ± 18.84 vs 74.05 ± 15.38; p-value = 0.041), SBP (134.65 ± 24.10 vs 122.40 ± 27.36)).

The estimation of eGFR considers multiple patient factors in its estimation. Creatinine, a parameter considered in the assessment of renal function based on glomerular filtration was established by the current study to have a statistically significant association with KD. The current study demonstrated that creatinine levels were elevated in patients with loss of kidney function as compared to their counterparts with normal renal function (102.15 (46.49) vs 67.53 (53.51); p-value = <0.001). Our findings come to add weight to studies by Kene *et al*. [35] whose research findings implicate a 43.2% serum creatinine impairment among T2DM patients. The Kene *et al*.’s [35] study had a population that was highly aged (<40=65 (22.7%); 40-49 = 40 (13.9); 50-59 = 82 (28.6); ≤60 100 (34.9)) and like that study, this current study has demonstrated elevated creatinine levels to be significantly associated with KD which could possibly be influenced by aging. Subsequently, the current study observed patient´s occupation and creatinine levels to be independently associated with KD. While the relevance of being employed has been shown to slow down the decline in the progression of kidney function [31], this study agrees and further establishes that being formally employed reduced the odds of KD. We associate such dynamics with a plethora of factors. These factors may include a steady source of income, insurance, and retirement packages, as well as access to money lending means in the purchasing of medications to manage the T2DM disease condition. On creatinine, Netere *et al*. [36] have associated the doubling time for creatinine in T2DM with the decline in renal function. Although serum creatinine was earlier implicated to be elevated in patients with KD, the current study has also established that a unit increase in creatinine across the varying groups of patients living with T2DM based on kidney functionality increases one´s odds (10% increased odds) of having a loss of kidney function hence supporting the grounds for monitoring of creatinine levels in T2DM for early detection of any decline in kidney function.

**Limitations of the study:** due to the retrospective nature of the study, certain variables that could have provided valuable insights into kidney function, such as dietary habits, exercise, and current medications, were unavailable. It was challenging to control for all potential confounding factors, as the original data may not have been collected with the current research question in mind. Furthermore, because the data was collected from past records, there may be biases in patient inclusion or exclusion, leading to an unrepresentative sample.

**Recommendations:** health researchers should prioritize investigating the potential adverse effects of T2DM medications on kidney function, as this represents a significant area for future exploration. Additionally, conducting a longitudinal study to determine the time frame within which kidney-related conditions manifest among T2DM patients in the study jurisdiction is essential. Such research will inform clinical decision-making by providing evidence-based guidelines for risk stratification, monitoring frequency, and intervention thresholds for individuals with T2DM. As a result, healthcare providers will be better equipped to tailor management strategies to individual patient needs and optimize outcomes.

## Conclusion

A significant majority of T2DM patients (70.2%) exhibited some degree of KD, with 1.4% progressing to kidney failure. The study identified employment status and creatinine levels as independent predictors of KD in this population. Given the high prevalence of KD among T2DM patients and the associated costs of managing the condition, it is essential to implement comprehensive preventive strategies. These strategies should include routine kidney function screening and targeted educational initiatives to promote kidney health. Special attention should be given to unemployed, informally employed, and retired individuals, as they may be at increased risk due to socioeconomic factors. Educational campaigns should aim to raise awareness about the importance of regular monitoring of kidney function, understanding the implications of elevated creatinine levels, and adopting lifestyle modifications that can help maintain kidney health. By prioritizing these vulnerable groups, healthcare providers and policymakers can mitigate the burden of kidney disease, improve patient outcomes, and reduce healthcare costs associated with managing advanced kidney disease.

### What is known about this topic

- About two-thirds of T2DM patients experience kidney dysfunction;
- In developing countries, T2DM patients are at a particularly higher risk of developing kidney dysfunction compared to those in developed countries, and in the African setting, the prevalence of kidney dysfunction among T2DM patients was found to be 22.0%;
- In Ghana, approximately 10% of deaths in people with T2DM are attributable to kidney failure.

### What this study adds

- This study reported a prevalence of 70.2% kidney dysfunction among T2DM patients, specifically, 43.3%, 3.5%, and 1.4% had mild kidney function, severe kidney function, and kidney failure respectively;
- This study identified employment status and creatinine levels as independent predictors for kidney dysfunction among T2DM patients.
